# Œstrus Ovis1Read at the meeting of the Pennsylvania State Veterinary Medical Association, September, 1899.

**Published:** 1900-01

**Authors:** J. F. Butterfield

**Affiliations:** South Montrose, Pa.


### ŒSTRUS OVIS.[1]

BY J. F. BUTTERFIELD, V.S.,
SOUTH MONTROSE, PA.

THE grub in the head of sheep is the larva of a gad-fly, œstrus ovis, which deposits the live embryo on the margin of the nostrils. From there it creeps up into the nasal sinuses and the sinuses of the head. It is often harmless, but sometimes causes much irritation, redness, and a white, muco-purulent discharge from the nostrils, with dulness and stupor, and even death from the congestion found near and in the brain.

I especially wish to call attention to the life-history of these parasites in the North. Most writers tell us the fly deposits the live worm during the summer months, and the mature larva makes its way from the head to the ground in spring, and there makes its change to the fly, to again lay another crop of embryo during the summer months. Heretofore it has been my experience to find the irritation caused by the grub to manifest itself during March and April.

The Pennsylvania State Live-stock Sanitary Board requested the writer to make an investigation into the cause of the loss of a number of sheep in the flock of Mr. R. S. Squires, of Lathrop, Susquehanna County. Mr. Squires had lost about fifty sheep in the past three years during the summer months from a flock of about 125. I visited Mr. Squires' farm about August 10, 1899, and found two that were presenting symptoms that others had shown which died. One was a two-year-old sheep, the other a four-months-old lamb. The symptoms present: Very thin in flesh, dull, sluggish, head drooped, and often held in a corner for hours, and a snotty nose. In the post-mortem examination the only alterations found were four or five of the œstrus larvæ in the sinuses of the head, with congestion of the coverings of the brain, especially the bony structure. Did not think we would find the grub of sufficient size to cause any trouble in the lamb, but upon an autopsy we found three larvæ in the sinuses of the nose that were three-fourths grown, and with the characteristic congestion in connection with the brain coverings. I find that one of the later investigators, Dr. Cooper Curtice, in *Animal Parasites of the Sheep*, tells us that in the vicinity of Baltimore, Md., he had

[1] Read at the meeting of the Pennsylvania State Veterinary Medical Association, September, 1899.

found in January very small œstrus larvæ in the heads of sheep, and he thinks the fly may appear at any time the temperature is not too low. These cases lead me to the same opinion.

As for treatment, my experience prompts me to think trephining and washing out with tepid water, after the larvæ have gained entrance to the sinuses of the head, is the only successful one, and that must be done before the sheep shows too much dulness or stupor. Where the larvæ are in the sinuses of the nose a solution of alum water injected into the nostrils seems to give them (the grub) most uneasiness. I have placed them in turpentine for half an hour, and they were as lively as ever. As for preventive treatment, smear the nose with a mixture of pine-tar and fish-oil, which can be done by salting from auger holes smeared around with the mixture. Also let the sheep in the cold dark basement of the barn during the heat of the day, tacking sticky fly-paper on the sides of the walls near the doors where the fly would naturally alight, thereby catching them before they deposit the little larvæ. This fly-paper can be very cheaply prepared by melting together two parts by weight of resin and one of castor-oil and smearing upon thick paper while warm.

